# Development of a Novel Home-Based Exergame With On-Body Feedback: Usability Study

**DOI:** 10.2196/38703

**Published:** 2022-12-06

**Authors:** Alexandra Schättin, Jordan Pickles, David Flagmeier, Benjamin Schärer, Yanick Riederer, Stephan Niedecken, Stefan Villiger, Roman Jurt, Nicole Kind, Sam N Scott, Christoph Stettler, Anna Lisa Martin-Niedecken

**Affiliations:** 1 Sphery Ltd Zurich Switzerland; 2 Department of Diabetes, Endocrinology, Nutritional Medicine and Metabolism Bern University Hospital University of Bern Bern Switzerland; 3 Department of Design Institute of Design Research Zurich University of the Arts Zurich Switzerland; 4 Team Novo Nordisk Professional Cycling Team Atlanta, GA United States

**Keywords:** exergame, iterative design, home-based exergame, on-body feedback, usability, training experience, gameplay experience, home-based exercise, serious games, physical activity

## Abstract

**Background:**

With more than 1.4 billion adults worldwide classified as physically inactive, physical inactivity is a public health crisis leading to an increased risk of cardiometabolic diseases. Motivating and engaging training strategies are needed to tackle this public health crisis. Studies have shown that exergames, games controlled by active body movements, are potentially usable, attractive, and effective tools for home-based training. The ExerCube (by Sphery Ltd) has been developed as a physically immersive and adaptive functional fitness game. The development of a home-based version of the ExerCube could increase accessibility, reduce barriers to exercise, and provide an attractive solution to improve physical and cognitive health.

**Objective:**

The aim was threefold: (1) to develop a usable home-based exergame system, (2) to evaluate the usability and training experience of the home-based exergame and its early-stage on-body feedback system, and (3) to identify avenues for further user-centered design iterations of the system.

**Methods:**

A total of 15 healthy participants (mean age 25, SD 3 years) completed 2 laboratory visits consisting of four 5-minute exergame sessions. In each session, the on-body feedback system provided a different feedback modality (auditory, haptic, and visual feedback) to the participant. Following the second visit, participants completed a range of assessments, including the System Usability Scale (SUS), the Physical Activity Enjoyment Scale (PACES), the Flow Short Scale (FSS), the Immersive Experience Questionnaire (IEQ), and a rating of perceived exertions (RPEs) both physically and cognitively. Participants answered questions regarding the on-body feedback system and completed a semistructured interview.

**Results:**

Usability was rated as acceptable, with a SUS score of 70.5 (SD 12). The questionnaires revealed medium-to-high values for the training experience (FSS: 5.3, SD 1; PACES: 5.3, SD 1.1; IEQ: 4.7, SD 0.9. Physical (mean 4.8, SD 1.6) and cognitive (mean 3.9, SD 1.4) RPEs were moderate. Interviews about the on-body feedback system revealed that the majority of participants liked the haptic feedback and the combination of haptic and auditory feedback the best. Participants enjoyed the distinct perceptibility, processing, and integration of the exergame and its supportive and motivating effect. The visual feedback was perceived less positively by participants but was still classified as “potentially” helpful. The auditory feedback was rated well but highlighted an area for further improvement. Participants enjoyed the training experience and described it as motivating, interactive, immersive, something new, interesting, self-explanatory, as well as physically and cognitively challenging. Moreover, 67% (n=10) of the participants could imagine exercising at home and continuing to play the exergame in the future.

**Conclusions:**

The home-based exergame and its early-stage on-body feedback system were rated as usable and an enjoyable training experience by a young healthy population. Promising avenues emerged for future design iterations.

## Introduction

Physical inactivity, defined as levels of physical activity lower than those generally recommended for optimal health as well as disease and premature death prevention [1], is a public health challenge [2], with more than 1.4 billion (~1 in 4) adults worldwide classified as physically inactive [3]. A number of common barriers to exercise, including a perceived lack of time, lack of motivation, financial restrictions, limited access to facilities, and appropriate equipment, as well as environmental factors such as bad weather, have been linked to low physical activity levels [4,5]. Home-based exercise has the potential to overcome some of these barriers to exercise, as it alleviates the need for travel and, if done indoors, overcomes the barrier of bad weather. The COVID-19 pandemic has accelerated this trend, with many people exercising at home due to the closure of sports facilities [6].

To date, many home-based exercise studies have focused on simple body weight exercises, resistance banded training, and traditional training methods (ie, walking and jogging) [7-12]. While these approaches are generally effective at improving markers of health, they may be hampered in their effectiveness due to motivational issues interfering with regular exercise. New, engaging, and effective strategies may help to overcome such common exercise barriers. Furthermore, these strategies have the potential to increase the physical activity level to the World Health Organization recommendation of at least 150-300 minutes of moderate intensity or 75-150 minutes of vigorous intensity physical activity per week, or an equivalent combination [13], in an increasingly inactive population.

Exercise games (exergames) concurrently combine physical and cognitive training through an integration of physical exercises within video games [14]. Exergames have been shown to improve both cognitive and physical health [15-19] while providing a more attractive exercise setting compared to traditional gym-based exercises [17,18,20,21]. Regarding the integration and use of exergames at home, the literature shows that exergaming in a home-based setting elicits high training adherence and compliance [22-24]. Furthermore, studies showed acceptable to high rates for feasibility and usability in terms of home-based exergames in children and older adults [22,25]. Moreover, results demonstrated favorable emotional experiences as well as positive responses regarding motivational aspects [22,23]. Regular exergame system updates, including further attractive games, could help to keep the experience and motivation at a high level [25]. In addition, exergaming was proposed to be an effective training approach to maintain physical activity at home during the COVID-19 pandemic [26]. Nevertheless, literature points out the importance of flawless technical functionality [22], as error-prone systems can exceed external support and lower training motivation [25]. Moreover, social and clinical guidance may remain necessary for sustained exergame engagement at home in certain settings [27]. Overall, exergaming has the potential for an attractive and effective home-based training strategy.

The ExerCube (Sphery Ltd), a physically immersive mixed-reality functional fitness game [21,28], has been found to provide an equally enjoyable and motivational experience when compared to traditional training methods [21,29]. Within the ExerCube, players are surrounded by 3 walls, which serve as projection screens and a haptic interface for energetic bodily interactions. A customized motion tracking system follows players’ movements. The ExerCube incorporates technology that adapts the game in real time to the individualized requirements through changes in training intensity and in-game difficulty based on continuous heart rate (HR) tracking and the user’s in-game performance [29,30]. The potential for the ExerCube as a feasible and effective exergame has been corroborated before [21,29,30]. However, the stationary version was originally designed for use within a gym-based environment and not for use in home-based environments.

To make the ExerCube available to a broader population, thereby reducing major exercise barriers, the development of a home-based version of the ExerCube would be advantageous. Therefore, the aim of this study was threefold: first, to develop a usable home-based exergame system by translating the spatial guidance and the motivating haptic feedback of the stationary version of the ExerCube into an exergame setting for home use; second, to evaluate the usability and training experience of the home-based exergame and its early-stage on-body feedback system; and third, to identify avenues for further user-centered design iterations of the system.

## Methods

### Design Process of the On-Body Feedback System

**Figure 1:**
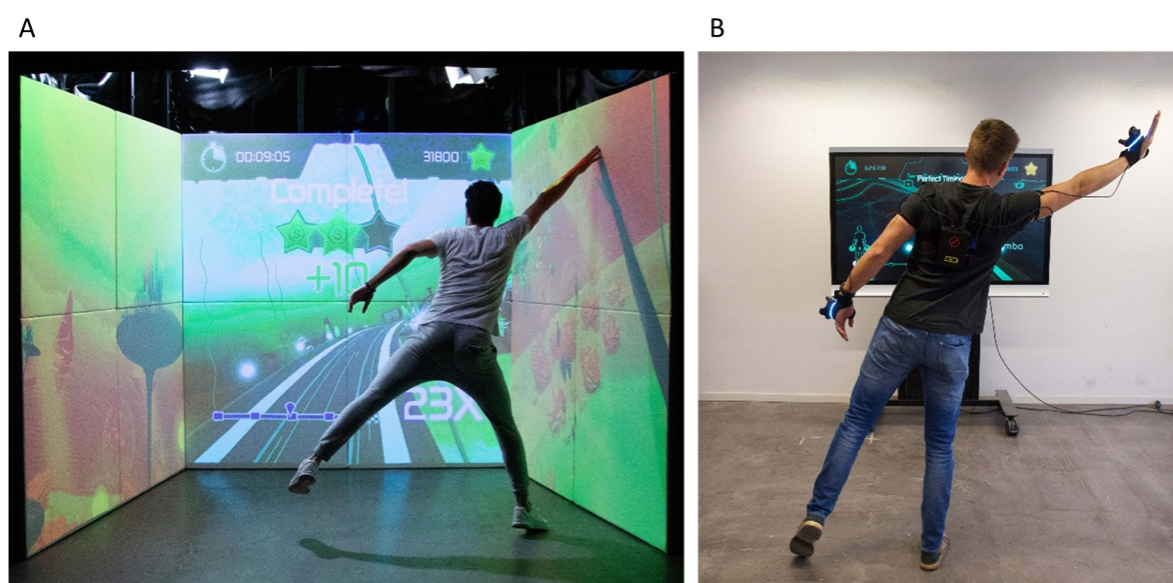
Comparison of the original full-size ExerCube and the first iteration of the home-based version. (A) The original ExerCube setting with the 3 walls. (B) The home-based exergame and its early-stage on-body feedback system (gloves). Note that in both versions, the user has sensors on the wrists and ankles so that movements are followed by the HTC Vive trackers. In the home-based version, feedback is provided either through auditory, haptic, or visual stimuli or through a combination of these modalities.

In an iterative, user-centered design process, an immersive on-body feedback system was developed to preserve the benefits of the physically immersive setup of the ExerCube (Figure 1A) for use in the home (Figure 1B). The first phase of the design process was to analyze the existing stationary exergame system and determine its technical opportunities, focusing on the attributes allowing for an immersive training experience. The most important finding was that the haptic and spatial experience provided by the 3 walls in the ExerCube had to be translated into an on-body feedback system. The focus was on using existing hardware as well as the player’s body to implement an additional feedback system.

Following a rethinking process, different concepts for the on-body feedback system were elaborated by industrial designers and sports scientists. To ensure that these concepts were user-centered, a focus group session was conducted with 5 young adults who were potential target users of a home-based exergame. The aim of the focus group interview was to explore the target group’s previous experiences with exergames; to define needs, preferences, and expectations for an optimal exergame experience; and to provide feedback about the elaborated on-body feedback systems using a semistructured interview guideline. The semistructured interview guideline was developed based on questions about home-based exergaming and especially on extending the gaming experience with an on-body feedback system.

Based on the feedback from the focus group session, the first version of the home-based exergame featured feedback gloves that were developed to provide the spatial perception and motivating haptic feedback needed to interact with the game. Vibration motors were used to resemble the haptic sensation of touching and punching without the need for physical walls. Visual and auditory feedback through light and sound were implemented as additional modes of interaction, exploiting the immersive experience of the game.

### Usability Study

#### Study Design and Procedure

**Figure 2:**
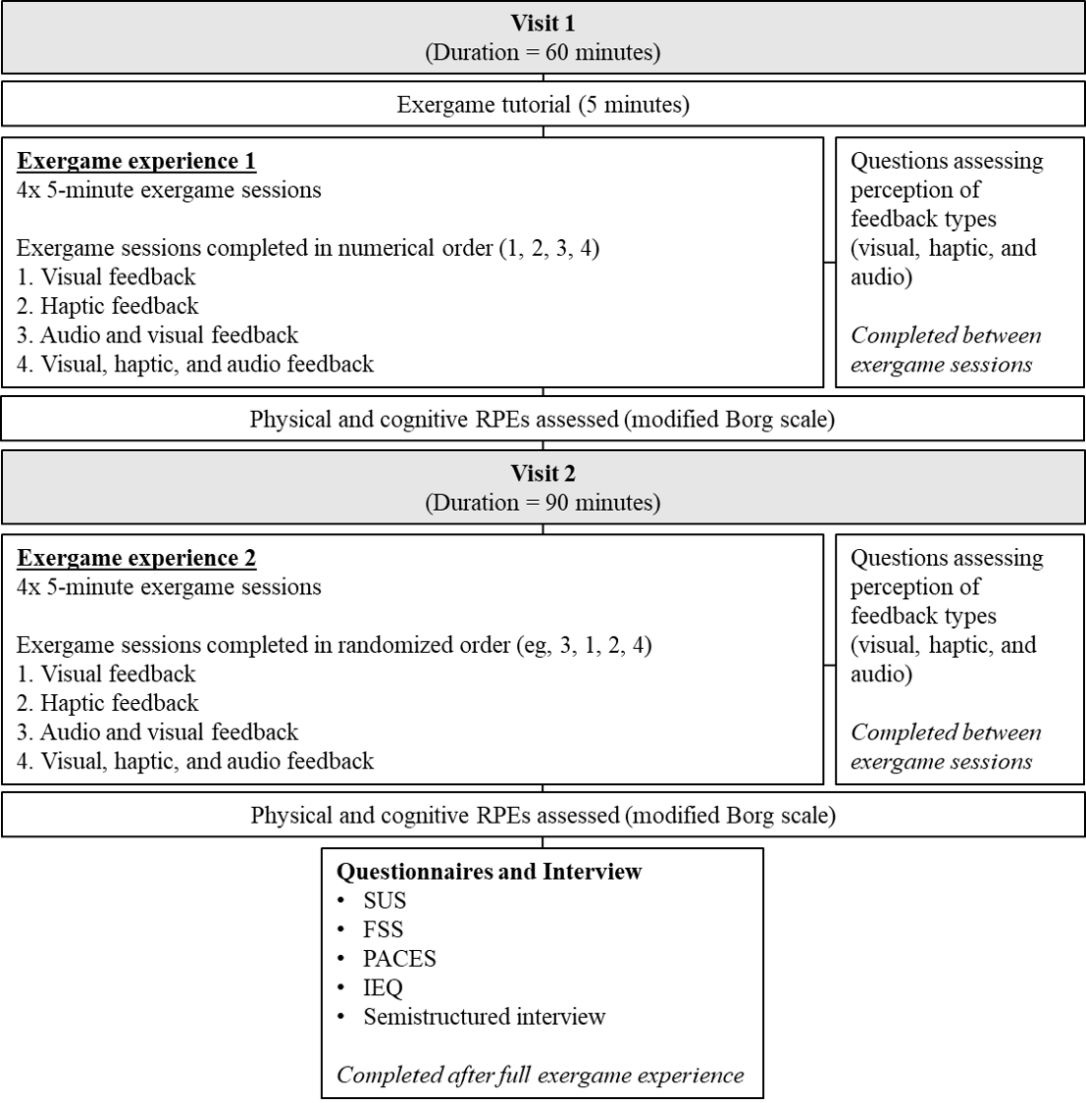
Protocol overview. Visual: light response on the hand sensor for correct movement; haptic: vibration response on the hand for correct movement; auditory: sound response for correct movement. FSS: Flow Short Scale; IEQ: Immersive Experience Questionnaire; PACES: Physical Activity Enjoyment Scale; RPE: rating of perceived exertion; SUS: System Usability Scale.

Healthy adults (age ≥18 years) participated in this study. Participants provided informed consent before participating. Participants attended 2 visits at the Inselspital University Hospital, Bern, Switzerland. Visit 1 familiarized the participants with the exergame through an in-game tutorial that provided in-depth instructions on how to complete each physical movement correctly before participants performed the exercise in a short sample of the game. Participants then completed four 5-minute exergame sessions, with each session separated by approximately 2 minutes of recovery time. Each session provided a different feedback modality to the participants (ie, whether they received feedback via auditory, haptic, or visual stimuli; Figure 2). In visit 2, participants completed the same four 5-minute exercise sessions as in visit 1. However, the order in which exercise sessions were completed was random. In both visits, participants were asked questions after completing each individual exergame session to assess their perception of the on-body feedback system. Following completion of all exergame sessions within visit 2, participants were asked to complete a range of questionnaires to assess usability and their exergame training experience. Participants then completed a semistructured interview with a member of the research team to assess their overall exergame experience. All interviews were conducted by author BS to eliminate researcher bias.

#### Ethics Approval

This study was granted an exemption from ethical approval by the Cantonal Ethics Committee, Bern, Switzerland (REQ-2021-00061).

#### Description of the Home-Based Exergame Set-up

“Sphery Racer” (by Sphery Ltd), a single-player game experience designed for the ExerCube setting, was used as the game environment. The “Sphery Racer” asks players to progress along a fast-paced racetrack via an avatar on a hover board. The customized motion tracking system (HTC Vive trackers) transfers the player’s movements (based on a functional workout) onto this avatar and thus onto the virtual racing track. Along the race, players are challenged by obstacles that require whole-body, functional physical exercises (eg, squats, lunges, and burpees) and by an additional cognitive challenge that includes quick information processing by deciding which exercise has to be performed when (ie, reaction time and coordinative challenges). Each correctly performed and timed exercise is rewarded with points. Players can advance through 5 game levels. Each level can be reached by performing a certain number of correctly executed physical exercises in succession (combo). Players are also demoted if they make mistakes. The higher the level, the higher the game speed (ie, the quicker appearance of the physical exercise). The greater the physical and cognitive challenge, the greater the point multiplier. To further ensure an ideal workout experience, the home-based exergame incorporates technology that adapts the game speed to the individualized requirements based on continuous HR tracking, aiming to keep the players at 80% of their maximal HR.

The newly developed on-body feedback system was integrated into the gloves of the tracking system to provide spatial guidance and motivating haptic feedback through different on-body feedback modalities. Flashing lights (visual feedback), vibration (haptic feedback), and sound (auditory feedback) appeared (depending on the predefined setting, Figure 2) when participants performed the correct exercise in the correct time window. The device illuminated red (visual feedback) when participants committed an error (wrong exercise or incorrect timing). Furthermore, the color of the lights changed depending on the game level (level 1: very dark cyan; level 2: dark blue; level 3: dark cyan-lime green; level 4: moderate orange; level 5: dark magenta).

#### Assessments of Usability and Exergame Training Experience

Exergame usability was measured using the System Usability Scale (SUS; α=.91) [31,32], consisting of 10 items rated on a 5-point Likert scale (0: not true; 4: true). The overall usability score was calculated and multiplied by 2.5 to give a score out of 100. A score of at least 70 was set for an “acceptable solution” in this study; between 50 and 70 was “marginally acceptable”; and below 50 was “unacceptable” [32,33]. Participants’ exergame training experience was evaluated through the Flow Short Scale (FSS; α=.90) [34]. The FSS consisted of 13 items rated on a 7-point Likert scale (1: not at all; 7: very much) and was measured in 4 dimensions: overall flow, fluency of performance, absorption by activity, and perceived importance. For the dimensions of overall flow, fluency of performance, and absorption by activity, scores closer to 7 signal a positive score. For perceived importance, a score closer to 1 is deemed a positive score. Participants’ enjoyment of the exergame training was assessed using the Physical Activity Enjoyment Scale (PACES; α=.96) [35-38], which consists of 18 bipolar statements with 7 points between statements. To assess immersion within the game, participants completed the Immersive Experience Questionnaire (IEQ), which consists of 31 items assessed on a 7-point Likert scale (1=strongly disagree; 7=strongly agree) [39]. The IEQ analysis categorizes the responses into the following subcategories: total immersion, challenge, control, real-world dissociation, emotional involvement, and cognitive involvement [39]. The final single-item measure of immersion was measured on a 10-point scale (1: not at all immersed; 10: very immersed). To assess both physical and cognitively perceived exertion, a modified 10-point Borg scale (1: very, very light; 10: extremely heavy) was used [40]. Finally, a semistructured interview was designed to provide a specific evaluation of usability and training experience. The interview consisted of questions in several categories: overall, game software, game control, at-home use, focus, motivation, training (physical and cognitive), and further thoughts, including wishes and ideas.

### Data Analysis

Quantitative data (questionnaires and ratings of perceived exertion scales) were analyzed and reported descriptively. The semistructured interviews were assessed by 3 of the authors (AMN, AS, and YR). The authors followed an iterative thematic coding approach (overall experience, game [software], game control [hardware], at-home use, focus, motivation, training, and further thoughts including wishes and ideas) based on qualitative content analysis [41]. The themes for the coding were based on the categories of the semistructured interview that were determined before the study started. For all interviews, the coders individually transcribed and coded the data according to the categories of the interview guidelines. In iterations, the coders discussed the emerging results until an agreement was reached. Finally, 2 of the authors (AMN and AS) further summarized the results to compile the individual statements into main findings.

## Results

### Participants

A total of 16 participants (n=9 females, n=7 males; mean age 25, SD 3 years) were recruited for the study. One participant did not complete visit 1 due to a technical issue (a connection issue with the battery source of the on-body feedback system); therefore, 15 participants (n=9 females and n=6 males; mean age 25, SD 3 years) completed both study visits.

### Questionnaire Responses

The data from the questionnaires are presented in Table 1.

**Table 1:** Quantitative data assessing system usability, flow, enjoyment, immersion, and perceived physical and cognitive exertion (N=15).

Measurement					Mean (SD)			Median (IQR)			Range
**System usability**											
	System Usability Scale			70.5 (12.0)			70.0 (65.0-77.5)			47.5-92.5	
**Training experience**											
	**Flow Short Scale**										
		Overall flow	5.3 (1.0)			5.6 (5.0-6.0)			2.8-6.3		
		Fluency of performance	5.5 (1.1)			5.8 (4.9-6.2)			3.3-6.8		
		Absorption	5.0 (1.2)			5.3 (4.9-5.6)			1.8-6.5		
		Perceived importance	2.3 (1.3)			2.0 (1.2-2.7)			1.0-6.0		
	Physical Activity Enjoyment Scale			5.3 (1.1)			5.6 (5.0-6.0)			2.3-6.5	
	**Immersive Experience Questionnaire**										
		Total immersion	4.7 (0.9)			5.0 (4.4-5.2)			1.7-5.4		
		Challenge	4.8 (0.9)			4.8 (4.5-5.4)			2.5-6.0		
		Control	4.5 (1.2)			4.6 (4.1-5.2)			1.0-5.6		
		Real world dissociation	4.3 (1.1)			4.3 (4.0-4.9)			1.1-5.4		
		Emotional involvement	4.0 (1.3)			4.0 (3.8-4.9)			1.0-5.8		
		Cognitive involvement	5.4 (1.3)			5.7 (5.2-6.1)			1.6-6.7		
		Immersion reliability check^a^	7.0 (2.0)			8.0 (6.3-8.0)			1.0-9.0		
**Rating of perceived exertions**											
	Physical			4.8 (1.6)			5.0 (4.0-6.0)			1.5-7.0	
	Cognitive			3.9 (1.4)			4.0 (3.0-5.0)			1.0-6.0	

^a^n=14. Scores presented as mean values, median values, SDs, IQRs, and the minimum and maximum values for each measurement.

### Interview Data: On-Body Feedback System

Questions about the on-body feedback system revealed that during visit 1, participants did not notice the flash and colors in the visual feedback mode. However, the red-colored lights, which appeared when an error occurred, were strongly perceived by the participants. The vibration (haptic feedback), as well as the timing of the introduction of this feedback in the game, were immediately registered by the participants. The vibration was perceived as supportive. If the vibration was perceived as distracting, it was only at the beginning, as participants became familiar with the game. The sound (auditory feedback) was strongly perceived. However, the sound was not perceived as pleasant, and the quality was criticized. Many participants preferred the combination of sound and vibration. Few preferred the version with vibration only.

After visit 2, most of the participants recognized when feedback was added or omitted. Almost half of the participants chose haptic feedback (vibration) alone as their favorite feedback, as it was perceived to be discreet and less distracting. Likewise, almost half of the participants preferred the combination of vibration and sound due to the more pronounced feedback and motivating effect. The changes in colors depending on the level were better classified in the second study visit. The flash was not recognized correctly because it was mostly out of the field of view.

In visits 1 and 2, the colors and the flash were neither rated as supportive nor as a distraction. The vibration was perceived very positively by all participants and was not distracting. The use of the sound feedback was also immediately registered by all participants. However, the sound was perceived as too loud by almost half of the participants. Likewise, the sound was described as rather unsupportive due to its poor quality, delayed sounding, and volume. Many participants were even distracted by the sound during the game for the same reasons.

Regarding the attachment of the on-body feedback system with straps on the wrist, about half of the participants found them not annoying, while the rest found this form of attachment rather less comfortable due to sweat, cables, and a battery backpack during exercises.

### Interview Data: Overall Training Usability and Experience

#### Overall Experience

Overall, participants enjoyed the study as well as the gaming and training experiences. They experienced positive feelings during the training and described it as *something new*, *fun*, *innovative*, *cool*, *interesting*, *motivating*, *immersive*, *entertaining*, *interactive*, *intuitive*, *clear*, *simple*, *effective*, and *challenging*.

#### Game Software

Most of the participants liked the game (*design*, *dive into another world*, *forget the training aspect*, *new concept*, and *different movements*). Some participants stated that they would suggest adjustments to certain features of the game (*one-sided movements*, *upper body relatively little involved*, and *repetitive exercises*). Furthermore, the game was comprehensible for the majority of the participants (*the game explained what needs to be done*, *at the second training it was already easier*, and *the tutorial explained the exercises*). Participants mentioned aspects to improve the comprehensibility of the game: *timing of the exercises*, *registration of the executed exercises*, and *explanation of the movement execution*. The video game environment and the narrative framework were positively rated or even not noticed by the participants while performing the exergame. Some participants would like to have more variety through alternative game scenarios (eg, *more realistic game environment*) in the future. Furthermore, all the participants noticed that the game adapted to their individual performance skills (speed of the game).

#### At-Home Use

Participants could imagine exercising at home with the exergame system (*little time and material*, *lockdown [COVID-19]*, *bad weather*, and *for people who do not want to train exposed*). They would install it in their living room, basement, or office and would want to spend a maximum of 5.7 (SD 3.8) minutes installing the system, suggesting that the game should be quick and easy to set up. For many of the participants, their existing TV at home would be big enough to play the exergame at home. Certain aspects were mentioned as reasons why they would not want to use the system at home: *difficult to set up*, *room size*, and *preference to do sport in nature* (eg, *jogging in the forest*).

#### Focus

All participants focused on the game during the training (*upcoming exercises*, *exercise execution*, *reaching a high score*, *game stimuli*, and *being absorbed by the gaming environment*) and paid less attention to what was happening around them. Few of them paid attention to their body during the training (*inconsistent registration of the movement and position in the room*). The majority of the participants felt immersed in the gaming environment. Some of them felt the immersion was interrupted due to *inconsistent exercise registration of the exercises* or *a delay in the game*. A few participants felt dissatisfied with the current setup and would prefer virtual reality (VR) glasses. Most of the participants were distracted (to varying degrees) from the training intensity by the gaming environment.

#### Motivation

Participants were motivated to move and get better in the game (*flow feeling*, *high score*, *gaming factor*, *correct movement execution*, and *high combo*)*.* Many of the participants stated that they would be more motivated to train with the exergame than visit a gym.

#### Training

Furthermore, the majority of the participants felt physically and cognitively challenged by the exergame (*upcoming exercises*, *more in the first training session*, and *the faster or more strenuous the game became the more you had to think*) but not to their limits. Participants identified the following aspects as their biggest challenges: *correct exercise execution*, *timing* (*when the game became fast*), *keeping concentration high*, and *switching from one side of the room to the other side*). For some participants, the movements felt natural, and for others, the movements felt partly natural (*jogging in place was weird* and *execution of the lunges*). Participants would train a maximum of 2.5 (SD 1.0) times per week (range 1-4 times per week) for a maximum of 30.0 (SD 11.7) minutes (range 10-60 minutes) with the fitness exergame.

#### Thoughts Including Wishes and Ideas

Finally, participants would like to continue to play the fitness exergame in the future, and thus they wished for further extension to keep training motivation at a high level over a longer period: a tutorial with a *more detailed description of the exercises*; *smaller, handier, and more reliable* game controller; *options* regarding feedback selection and the video game environment; as well as *more and different movements for the training*.

## Discussion

### Principal Findings

The aim of this study was threefold: first, to develop a usable home-based exergame system by translating the spatial guidance and the motivating haptic feedback of the stationary version of the ExerCube into an exergame setting for home use; second, to evaluate the usability and training experience of the home-based exergame and its early stage on-body feedback system; third, to identify avenues for further user-centered design iterations of the system. Study results showed that the home-based exergame and its on-body feedback system were usable for a young healthy population. Regarding the on-body feedback system, participants enjoyed the haptic feedback and the combination of haptic and auditory feedback the best due to their supportive and motivating attributes. Furthermore, questionnaires and the semistructured interview indicated that participants enjoyed the motivating, interactive, and immersive gaming and training experience. Moreover, findings for future iterations of the home-based exergame system emerged from this evaluation process.

### Usability of the Exergame and its Feedback System

The SUS score of 70.5 (SD 12) is an acceptable score according to Bangor et al [32,33] and meets the “acceptable solution” value (set at least 70) of this study. Next to the SUS, the system’s usability was also reflected in the participants’ interview responses. Participants quickly understood how to control the exergame and rated it as self-explanatory and comprehensive. Furthermore, participants enjoyed the experience with the prototype on-body feedback system and could imagine using this exergame at home. The result of this study is on par with the literature, showing that participants found exergames to be an acceptable and usable solution for training in a home setting [22,25]. Thus, the integrated iterative user-centered design process seems to be an essential aspect of designing usable exergames [42,43].

Nevertheless, certain aspects were mentioned in the interview that would further improve the system’s usability and, thus, the gaming experience. These suggestions included analogue (eg, floor markings) or digital (eg, in-game visual or auditory signals) reference points to define the training area for the player. In the stationary ExerCube, the player is surrounded by 3 walls defining the training area. The challenge for future development work is to define the training area in a way that maintains the training experience and flow at a high level for the player. A further challenge that is connected to the training area is the timing of the exercises, meaning when the player has to initiate the movement to meet the target and reach the full score. In the ExerCube, the targets are moving from the front wall to the side wall. Thus, the home-based system should have alternative information that supports the player in deciding the timing of the movement. Another challenge that goes along with the space aspect is the training room, as each user has different space conditions at home. Therefore, the home-based system should have a certain degree of flexibility (eg, via calibration) to adjust to different room situations. Furthermore, the exergame should include further information and explanations of the executed movements. This aspect is even more important for home-based exergame training compared to training in a fitness class that is usually supervised by an instructor.

Regarding the on-body feedback device, the system has to be further developed and integrated into the existing devices (eg, tracking system), allowing the exergame to work as one complete system. This step should increase the usability of the system. Moreover, the visual and auditory feedback have to be improved in order to provide an additional supportive system for the above-mentioned challenges of an at-home exergame system. Furthermore, the feedback should be better integrated into the existing exergame scenario, providing the player with an on-body projected gaming experience. Overall, the study showed that the home-based exergame, as well as its early-stage on-body feedback system, is a usable training system for young healthy adults. Promising avenues and challenges were uncovered that have to be faced in future R&D work to further improve the usability and training experience of the home-based exergame.

### Game and Training Experience

The interview results showed that the participants enjoyed the study as well as the training experience. The majority of the participants reported that they felt immersed in the gaming environment and were motivated to move and get better at their game performance. Alongside the interview results, the questionnaire data (FSS, PACES, and IEQ) showed medium-to-high scores, indicating that participants were immersed in the gaming environment, enjoyed the training, and experienced flow feelings during the exergame training sessions. Flow and immersion, especially those generated by exergame training approaches, can divert the player’s attention away from the physical effort [29,44-46]. In terms of training motivation, this training experience and the shift in focus could be crucial to increasing intrinsic training motivation and, thus, long-term training motivation and adherence in physically inactive people [46,47]. These findings are in line with previous studies showing that home-based exergames can elicit favorable emotional experiences as well as positive responses regarding motivational aspects [22,23].

Moreover, the exergame training approach has to have an individually effective training load to experience flow [48,49] as well as trigger physical and cognitive improvements when performed over a longer time period. The assessed perceived exertion values showed moderate intensities for the physical and cognitive aspects. This is in line with the interview results, which showed that the participants felt physically and cognitively challenged by the exergame. The World Health Organization recommends at least 150-300 minutes per week of moderate intensity physical activity or 75-150 minutes per week of vigorous intensity physical activity, or an equivalent combination [13].

Overall, the results demonstrated that this exergame and its early-stage on-body feedback system have the potential to be an attractive home-based training solution. The newly developed system triggered similar game and training experiences as shown in previous studies using the ExerCube [21,29,30]. Furthermore, this home-based exergame solution could overcome identified barriers to exercise, such as lack of time, limited access to facilities and appropriate equipment, bad weather, and lack of motivation [4,5], and thus increase the activity level of physically inactive people [47,50]. Nevertheless, further extensions and variety of the exergame might help to maintain motivation as well as the training load at an optimal level over longer training periods in players using the system at home [25]. 

### On-Body Feedback System

The interview data revealed that participants enjoyed the experience with the on-body feedback system. The haptic feedback, in particular, was well received as a single stimulus as well as in combination with the visual feedback. This is in line with findings from previous studies in exergames, which showed that haptic feedback positively influences the feelings of spatial presence, immersion, flow, and motivation [21,51,52] as well as embodiment [53].

Similar to other studies, it could be shown that haptic stimuli in exergames are preferred over auditory and visual feedback [54,55]. In our study, this might also be amplified by the fact that the visual and auditory processing loads were already quite high because of the new information from the virtual game scenario, and thus players had more resources for the haptic feedback. Therefore, the cognitive resources for the auditory and visual feedback remain limited, at least during the first gaming sessions. It can be assumed that this will change after several gaming sessions are completed.

Generally, the features of the newly designed on-body feedback system are very promising. However, the study provided inspiring avenues for further development of the on-body feedback system. Auditory feedback, for example, needs to be further optimized since there are specific requirements for auditory feedback and music in sports [56] and exergames [21].

In future research, it would be interesting to evaluate the impact of the different on-body feedbacks on the feelings of empowerment [57] and self-esteem [55] as well as on aspects of movement quality [58].

### Limitations

A limitation of this study is the lack of variability in the sample; as the sample within this study was all young healthy participants with a small range in age (19-30 years), the inclusion of a wider age range within this study would have increased the generalizability of the findings. Moreover, there was no defined washout period between the visits, meaning that participants had different time periods between visits 1 and 2. However, no participant had more than 2 weeks between visits; therefore familiarization was similar across all participants.

### Conclusions

Overall, the results showed that the home-based exergame and its early-stage on-body feedback system were rated as usable and an enjoyable training experience by a young healthy population. Results demonstrated that this exergame and its on-body feedback system have the potential to be an attractive home-based training solution. Furthermore, avenues emerged for future design iterations of the home-based exergame system. Future studies are needed to investigate the feasibility of the exergame in physically inactive people, assess participants’ experiences of repeated home-based use of the exergame, and examine preliminary training effects on physical and cognitive functions in young adults.

## Abbreviations

Exergame: exercise game
FSS: Flow Short Scale
HR: heart rate
IEQ: Immersive Experience Questionnaire
PACES: Physical Activity Enjoyment Scale
SUS: System Usability Scale
